# Sprengel's Shoulder

**Published:** 1909-02-20

**Authors:** 


### Surgery.

### SPRENGEL'S SHOULDER.

Congenital elevation of the scapula is commonly known in this country as Sprengel's shoulder. This observer published an account of four examples of the affection in 1891, but he was not the first to call attention to it, Eulenburg, in Germany, having preceded him in doing so by thirty years. Since that time some hundred and thirty cases have been recorded at different periods in medical literature. On these statistics one might be led to suppose that the deformity was one of great rarity, but for every case that is recorded, many unpublished ones exist, and a surgeon connected with the out-patient department of a children's hospital may come across several such cases in the course of a year. The child is not often admitted to the wards, either because the condition is causing no serious inconvenience (the mother bringing him upon account of his abnormal appearance), or because it is obvious that operative treatment is unlikely to benefit him.

As its name implies the deformity consists of a displacement of one or both scapulæ above the normal level. This is the most striking abnormality. But other departures from the normal are usually present. Thus the affected scapula is rotated on its sagittal axis, and is altered in shape so that its vertebral border is shorter on the affected side. In this respect the bone shows a retrogression to the quadrupedal type, owing apparently to weakness of the scapular muscles; the human type of scapula having been evolved to accommodate the increased musculature demanded by the free movements at the shoulder joint in man. The upper border is often found to be beaked or curved in a forward direction. This anomaly is best explained on the hypothesis that the elevation of the scapula is due to imperfect descent from its original cervical position in the fœtus, and the superior border is subsequently bent forward because the normal support of the posterior surfaces of the upper rib is absent. Finally, many of these bones present bony outgrowths which occasionally unite the scapula to one or other of the upper dorsal vertebræ. The origin of these outgrowths has given rise to much discussion. Some believe that the bony process originates in the vertebra and becomes secondarily attached to the scapula, while others hold the reverse view. In a few cases recorded the appearance and position of the bridge of union has been such as to cause it to be regarded as an ossified portion of one of the rhomboid muscles. The condition, like most congenital malformations, is rarely a single defect, but is usually associated with one or more concomitant deformities. The commonest of these are defects of the ribs, vertebræ, humerus or clavicle, and defects of the adjacent muscular system are frequently found, particularly of the trapezius pectoralis major and sterno-mastoid. In addition to these, isolated instances are on record of attendant deformities in a distant part of the body, *e.g.* talipes equinus, spina bifida, and even strabismus.

Children suffering from this deformity are brought up by their mothers who are anxious on account of the asymmetrical appearance. The neck on the affected side is fuller and shorter than the sound side, the muscles are tense and the chin may be drawn down on to the chest. The exact relation of the scapula to the trunk varies in each case. The only abnormality that is constant is its high position. But in addition to this it may be rotated round its sagittal or coronal axis, or may be farther from or nearer to the vertebral column than usual. So manifold are the minor variations from the normal that it is almost impossible to classify them.

The patient presents few actual symptoms, except that, since the mobility of the scapula is lessened, movements of the arm on the affected side will necessarily be restricted. In a genuine case, abduction beyond a right angle is not attainable. When the scapula is fixed in an abnormal position by a bridge of bone, the restriction of arm movement may be so advanced as to be a serious inconvenience.

Many hypotheses have been put forward to explain the causation of the condition. In most of them stress is laid upon the important part played by some modification of the normal intra-uterine conditions, *e.g.* lack of amniotic fluid (as the result of which the force of the uterine contraction is brought to bear directly upon the fœtus) or the presence of amniotic adhesions. Others again suppose that the scapula is deformed, as part of a general maldevelopment, and for this reason is retained in an abnormal position. The frequent association of other deformities, already mentioned, would seem to lend weight to this hypothesis. Whatever view is taken of the actual causation it would seem, however, that the scapula is not displaced upwards into an abnormal position, but that it is retained in its fœtal relations in extra-uterine life. The condition has some points of similarity with a retained testis in this respect.

The treatment depends on the nature of the case. Where the scapula is retained in an abnormal position by weakness, or ill-development of muscles which should normally pull it down, it may be sufficient to strengthen these muscles by special exercises and massage. Great improvement of the deformity can be obtained by use of these simple means alone. If, however, no benefit ensues, it is probable that there are counteracting muscle-bands or adhesions, and it is quite justifiable to undertake an operation for the purpose of dividing them. In those cases in which the scapula is united to the trunk by osseous bands no treatment can be of any avail, short of cutting down and dividing the bridge of bone. In considering the advisability of operation in any case, the extent of the deformity must be balanced against the severity of the proceeding. A favourable prognosis can rarely be given when the condition is associated with absence of ribs or marked scoliosis.

Those who are interested in this subject will find in the report of research work just issued by the division of surgery of Harvard University an admirable article by Dr. Horwitz, in which all the existing information on the deformity is lucidly reviewed.

